# The Fabrication and Characterization of BaTiO_3_ Piezoceramics Using SLA 3D Printing at 465 nm Wavelength

**DOI:** 10.3390/ma15030960

**Published:** 2022-01-26

**Authors:** Andrey Smirnov, Svyatoslav Chugunov, Anastasia Kholodkova, Maxim Isachenkov, Andrey Tikhonov, Oleg Dubinin, Igor Shishkovsky

**Affiliations:** Skolkovo Institute of Science and Technology, 30/1 Bolshoi Boulevard, 121205 Moscow, Russia; An.Smirnov@skoltech.ru (A.S.); S.Chugunov@skoltech.ru (S.C.); maxim.isachenkov@skoltech.ru (M.I.); a.tikhonov@skoltech.ru (A.T.); O.Dubinin@skoltech.ru (O.D.); i.shishkovsky@skoltech.ru (I.S.)

**Keywords:** 3D printing, BaTiO_3_, piezoelectric properties, Perovskites, additive manufacturing, piezoceramics, Stereolithography (SLA)

## Abstract

The additive manufacturing of BaTiO_3_ (BT) ceramics through stereolithography (SLA) 3D printing at 465 nm wavelength was demonstrated. After different milling times, different paste compositions with varied initial micron-sized powders were studied to find a composition suitable for 3D printing. The pastes were evaluated in terms of photopolymerization depth depending on the laser scanning speed. Furthermore, the microstructure and properties of the BT ceramic samples produced through SLA 3D printing were characterized and compared with those of ceramics fabricated through a conventional die semi-drying pressing method. Three-dimensional printed samples achieved relative densities over 0.95 and microhardness over 500 HV after sintering, nearly matching the relative density and microhardness attained by the pressed samples. Upon poling, the 3D-printed samples attained acceptable piezoelectric module d_33_ = 148 pC/N and dielectric constants over 2000. At near full density, BT piezoceramics were successfully fabricated through SLA 3D printing at 465 nm wavelength, achieving photopolymerization depth of more than 100 microns. This work paves the relatively low-cost way for 3D printing of piezoelectric ceramics using conventional micron-sized powders and high printed layer thickness.

## 1. Introduction

The traditional manufacturing methods are often limited in the complexity of the technologically achievable shape of ceramic items. When piezoceramic items of complex geometry are demanded, some of these methods are physically incapable of producing the necessary geometrical features, while other methods require costly molds and dyes [1] or involve complex processing after the sintering [2]. Furthermore, these processing machining methods often lead to microstructure distortion and surface damage, which post-machining annealing can often ameliorate. However, in some applications employing piezoelectric shear modes, the geometry demands that machining is carried out after poling, which rules out the possibility of annealing due to the high probability of the piezoelectrical properties degradation [3]. The use of additive manufacturing (AM) methods for piezoceramics production could overcome the mentioned adverse effects. In particular, the complex shapes of items with a slight increase in production cost, traded for good physical properties, become possible. This is especially critical for small-batch manufacturing [4]. The most commonly used AM methods of piezoceramic green parts manufacturing are Fused Deposition Modeling (FDM) [5], Selective Laser Sintering (SLS) [6], Ceramic Binder Jetting/Inkjet Printing (BJ/IJ) [7], Ceramic paste extrusion (Robocasting) [8], and Stereolithography (SLA)/Digital Light Processing (DLP) [9].

Photopolymerization-based AM, such as SLA/DLP, is a well-designed widespread technology applied to manufacturing of technical ceramics [10]. The physical principle of layer applications to the build platform and selective photopolymerization differs between SLA and DLP technologies. The SLA approach uses highly viscous paste as the base material, as well as UV laser, to selectively polymerize contours and shade the areas within the build platform. The DLP method uses relatively low viscosity suspensions as the base material and either a simple UV/Vis source of light projected to the entire build platform through a physical mask, or a Digital Mirror Device containing arrays of micromirror to selectively reflect the light from a light source to the build platform. Stereolithography-based ceramic AM often reach precision down to 30 µm within a layer and the layer thickness of 10–50 µm. The final resolution is even better due to the shrinkage of 3D-printed parts during sintering (on average, by 15–25%). The volume fraction of ceramic powder reaches 70–80 wt.% in pastes and 60–70 wt.% in suspensions. The higher volume fraction leads to lower shrinkage and higher final density of the ceramic product. A thermal processing stage is required for the 3D-printed green parts to burn off the organic binder and sinter the ceramic material.

Both technologies were applied to piezoceramics 3D printing with different success. The 3D printing of piezoceramics with photopolymerization-based technologies is, in general, similar to the 3D printing of regular ceramic materials [11]. Piezoceramic materials, such as BT, feature greater refractive indices when compared to the traditional technical ceramics Al₂O₃, ZrO₂, or yttrium stabilized zirconia (YSZ), that are successfully used in SLA and DLP technologies. When mixed with an organic binder, the high refractive index materials increase photon scattering during the UV/Vis exposure stage of the 3D printing process, efficiently increasing the polymerized area and decreasing the polymerization depth [12]. The polymerization depth decrease requires thinner layers (down to 10–20 μm) when 3D-printing the material [13]. As a result, a weak interconnect between the adjacent layers might happen, leading to delamination and in-layer fracturing during subsequent thermal processing of the 3D-printed items. In SLA/DLP methods, the 2–3 layers overlap of polymerization depth is typically provided during the photopolymerization to relieve these effects. The proper photopolymerization becomes an issue with thin layers, although the scientific community demonstrated some successful examples of such an approach.

Jang et al. [14] provided a fundamental understanding of BT-based ceramic suspension behavior under photopolymerizing conditions. They tested different incremental loadings (0–55 vol.%) of BT powder in a suspension. The suspensions behavior under UV curing was tested with an SLA 3D printer having a 350 nm laser. The authors mentioned that the cure depth of the suspensions decreases from 550 μm at 10 wt.% solid loading to below 250 μm at 30 wt.% solid loading when 100× repetitions of UV exposure were used. At a single exposure, the cure depth below 100 μm was observed for the best case, and the other cases were well below 50 μm. The authors attributed the poor polymerization of the BT suspensions to the significant differences in refractive indices of the ceramic powder and the organic binder that lead to excessive photon scattering. 

Chen et al. [15] demonstrated a 3D printing of BT piezoceramic samples using a hand-made digital mirrors-enabled DLP setup [13], and 70 wt.% solid loading of 100 nm BT powder. The authors did not mention the wavelength and power of the light source used for photopolymerization, exposure time, and layer thickness. The sintered at 1330 °C material had density of 93.7% of the theoretical maximum for BT, the dielectric loss tangent of 0.012 (versus 0.1 for typical BT), the ε (1 kHz) of 1350 (versus 1700 for typical BT), and piezoelectric module d_33_ 160 pC/N (versus 190 pC/N for typical BT). Song et al. [16] demonstrated another attempt to 3D print BT-based piezoceramic parts using digital-mirrors-enabled DLP technology. They formulated a few ceramic suspensions using 30–80 wt.% of 1 μm-sized BT powder. The cure depth of 180 µm was reached for 30 wt.% solid loaded suspension at 16 s of exposure time, while, for more practical 60–80 wt.% solid loaded suspensions, the best cure depth of around 50 µm was achieved at 8 s and 16 s of exposure time. The authors deduced from the preliminary test the best layer thickness of 20 µm at exposure time 2–8 s for 60–80 wt.% loaded suspensions. Measured properties of BT samples fabricated by digital-mirrors-enabled DLP process and sintered at 1330 °C were relatively low: piezoelectric module d_33_ = 87 pC/N, dielectric constant ε (1 kHz) = 920, dielectric loss tangent tan δ = 0.07.

Chen et al. [17] fabricated BT piezoelectric samples using the commercial DLP printer (Asiga max, NSW, Australia) with 385 nm wavelength. During the DLP process, the layer thickness was set as 10 μm for all the studied samples. To prepare stable and homogeneous BT suspensions, the 40 vol.% BT powder with the submicron powder particle size (200 nm, 500 nm, and 600 nm) and 60 vol.% photocurable resin. The maximum relative density of 0.98 was obtained in the samples made of 200 nm powder and sintered in the range 1300–1330 °C, while the best piezoelectric properties were recorded in the samples prepared from 600 nm powder: ε (1 kHz) = 4423, tan δ (1 kHz) = 0.019, d_33_ = 206 pC/N, relative density of 0.96. In Reference [18], LCD-SLA 3D printing method with 405-nm LEDs UV light source was used to create BT piezoceramic samples. Three types of BT powders were used: one with a particle size d_50_ = 3.4 μm, another BT powder with a particle size of d_50_ = 1.02 μm, and nanoscale BT powder with a particle size of d_50_ = 50–70 nm. The study showed significant delamination and spalling for samples prepared from micron-sized BT powder with d_50_ = 3.4 μm. No such defects were found for the ceramic slurries prepared from finer powders. The highest relative density (0.90) and piezoelectric properties (ε_r_ = 1965, d_33_ = 200 pC/ N, tanδ = 0.017) were measured in the sample sintered at 1300 °C with a dwell time of 4 h.

In the work of Cheng et al. [4], BT ceramics were manufactured based on the SLA 3D printing method. The authors do not mention the light source’s wavelength and power, exposure time, and layer thickness. The authors used a photosensitive resin with undisclosed components and a BT powder (particle size 500 nm) to formulate SLA-suitable slurry with powder loading: 70, 75, 80, 82, 84, and 86 wt.%. The BT ceramic samples sintered at 1290 °C exhibited acceptable piezoelectric properties: d_33_ = 166 pC/N at 80 wt.% BT powder. To evaluate the performance of 3D-printed BT piezoceramic samples, a 1.4 MHz focused ultrasonic array was fabricated and characterized. The 6 dB bandwidth of the array was at 40%, and the insertion loss at the central frequency was 50 dB. The results showed that the 3D-printed BT piezoceramic array has good potential as an ultrasonic transducer. Another promising example of using the SLA 3D printing technique for BT piezoceramics was shown in the work by Wang et al. [19]. The suspensions with 40 vol.% BT nanoparticles (mean particle size 500 nm) displayed shear thinning behavior and relatively low viscosity of 232 mPa·s at a 46.5 s^−1^ shear rate. The green BT samples fabrication was performed using a 3D printing device based on the bottom-up mode SLA process. The UV light source has a 405 nm wavelength laser providing a photopolymerization depth of 65 μm. After debinding and sintering at 1320 °C, the 3D-printed ceramic specimens showed a nanometer-level grain size and about 95% of the theoretical density, excellent dielectric properties (ε_r_ = 2726 and tanδ = 0.016 at 1 kHz), and acceptable piezoelectric constant, d_33_ = 163 pC/N.

In all the above mentioned studies, light sources with wavelengths of 350–405 nm were used, limiting the depth of photopolymerization [11]. In our previous work [20], it was shown that the change of the laser wavelength to 465 nm could significantly increase the efficiency and speed of piezoceramic paste photopolymerization. At the moment, no data could be found in the literature on the piezoelectric properties of BT ceramic samples obtained with SLA at a wavelength higher than 405 nm. In addition, there was no direct comparison of additive and conventional manufacturing results for the BT piezoceramics, when the same initial powder and sintering conditions were used. This study was aimed at conducting a comparative study of BT ceramics manufactured with SLA and traditional semi-dry pressing, to identify the correlations between powder particle sizes, piezoelectric properties, relative density, microhardness, and microstructure of the material.

## 2. Materials and Methods

**Figure 1:**
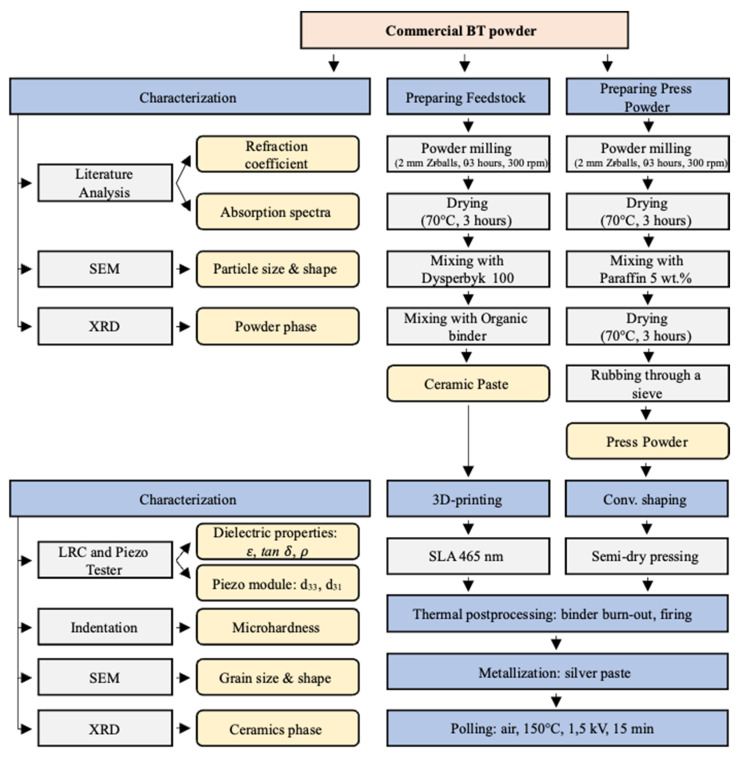
The diagram of comparative experimental study of BT powder behavior in additive SLA-based and conventional manufacture processes.

Based on the previous results [20], the 465-nm range for the laser source was selected for experimental testing of SLA-based BT ceramics manufacturing. A series of samples were prepared using SLA technique and the traditional method of semi-dry pressing. The samples were thermally treated in the high-temperature oven, all at once, using the same heating procedure. The characterization of the sintered samples was conducted in accordance with the workflow shown in Figure 1. As can be seen from the presented workflow (Figure 1), the number of steps in the additive route is one less than in the conventional one. 

### 2.1. Materials

A commercially available BT powder was purchased from a local supplier (LLC Aril, Ufa, Russia). The powder was ball milled in a planetary mill Pulverisette 6 (Fritsch, Idar-Oberstein, Germany) for 0 to 3 h in the presence of 3 mm zirconia balls and isopropyl alcohol to study the powder size influence on 465 nm SLA processes and BT ceramics properties. The powder was then kept in a furnace at 70 °C for 3 h to dry off the isopropyl alcohol. There were made 7 powder batches: (“as is”, milling for 15 min, 30 min, 45 min, 60 min, 120 min, 180 min). The pre-processed powder was characterized and used for experimental testing following the workflow shown in Figure 1.

### 2.2. SLA 465 nm 3D Printing

**Figure 2:**
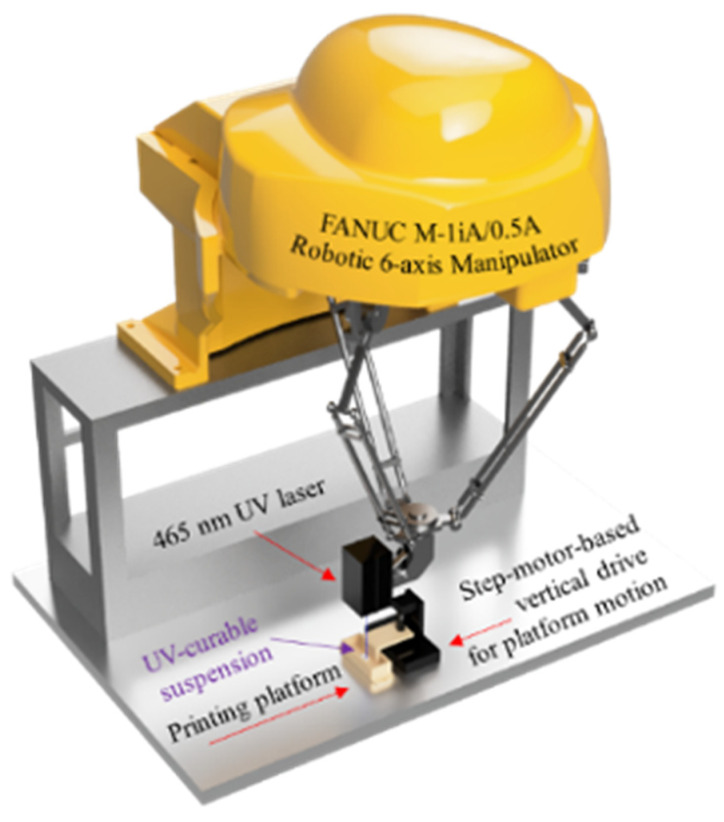
The image of a hand-made experimental 465 nm SLA laboratory setup.

The experimental SLA 3D printing setup (Figure 2) used an industrial 465 nm laser LDM450-3-12 (Purelogic R&D, Voronezh, Russia), originally designed for laser cutting and engraving operations. The laser was attached to FANUC M-1iA/0.5A (FANUC, Oshino, Japan) industrial robotic 6-axis manipulator to enable mechanical XY-motion of the laser beam. A vat and a printing platform were designed and printed from ABS plastic using an FDM 3D printer Ultimaker S5 (Ultimaker, Utrecht, Netherlands). The printing platform’s ascending and descending was implemented with the vertical mechanical drive based on a Nema17 Stepper motor KS42STH48-1684A (HANPOSE 3D Technology Co., Guangzhou, China). The printing operations with the experimental SLA setup were similar to those of the commercial SLA machine, except that the paste was applied and leveled by hands, while keeping the layer thickness of 100 μm. A detailed description of the experimental setup testing is given in our previous work [20].

After milling, the powder was mixed in a mill with a dispersant DYSPERBYK-100 (BYK-Chemie GmbH, Wesel, Germany). Next, the prepared powder was mixed with an organic binder, designed for SLA-based 3D-printing, purchased from a local supplier (Moscow, Russia), in a planetary mill Retsch PM400 Planetary (Retsch GmbH, Haan, Germany), at a speed of 300 rpm, for 2 h to achieve a good homogeneity. The resulting compositions were also placed in light-blocking jars. There were 7 configurations of ceramic paste with the maximum volume filling with powder BT (Table 1) prepared for testing the effect of powder sizes on process parameters and samples properties. The volumetric filling of the paste with BT powder depended on the particle size. The further increase of the solid content in the paste made it unspreadable. The samples were prepared in a shape of rectangular prisms with a side of 15 mm and height of 3 mm.

### 2.3. Semi-Dry Pressing

To compare the properties and microstructure of additive samples, disk-shaped samples with a diameter of 10–11 mm and a height of 2–3 mm were made by semi-dry pressing method. BT powders were used after milling, identical to those used for paste polymerization tests and obtaining 3D-printed samples. The composition of the press powder, the method of its preparation, and the molding modes during semi-dry pressing were selected based on the work results [21]. A solution of 5 wt.% of paraffin in petroleum was used to prepare the press powder. After introducing the binder, the mixture was dried in a furnace at 70 °C to a constant mass for the petroleum evaporation and rubbed through a sieve with a cell of 300 microns for powder deagglomeration. Further, the obtained press powder was uniaxially pressed by the hydraulic press at 100 MPa in the stainless-steel die with an internal diameter of 12 mm.

### 2.4. Post-Processing: Thermal Treatment, Metallization, Polling

**Figure 3:**
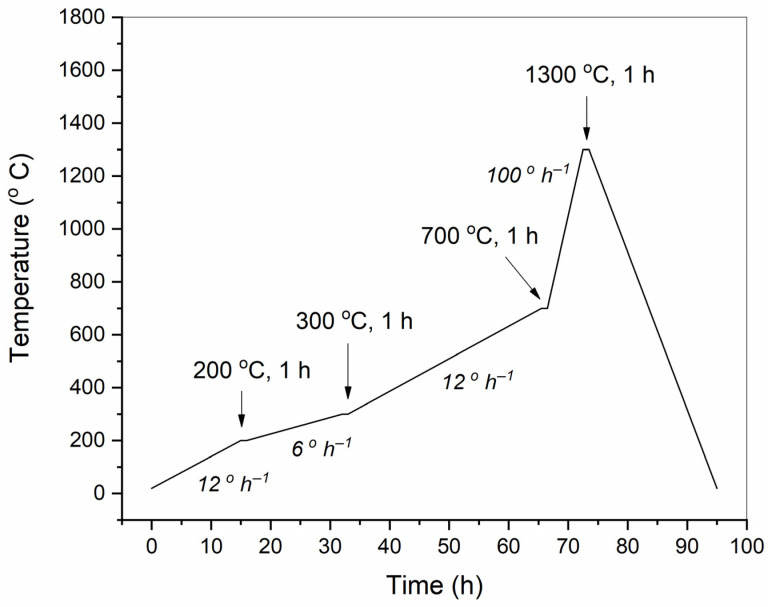
Temperature-time mode of binder burn-out and firing of BT ceramic samples. Arrows indicate dwell temperature and duration; heating rates shown by italic.

For a correct comparison of the properties and microstructure of conventional samples with 3D-printed ones, the binder burn-out and firing were carried out, together with additive samples, simultaneously, in a furnace. The temperature and time modes of binder burning and firing are shown in Figure 3. 

The polarization of BT ceramic samples was carried out in an air environmental oven using compressed air flow for samples and electrodes cooling, at a temperature of 120 °C, an electric voltage of 1500 V (pulse current), wherein exposure at temperature and voltage was 15 min, and then cooling to 60 °C under the electric voltage. All samples withstood the polarization voltage without damage.

### 2.5. Measurement of Powder and Samples Properties

The milled powder particle size distribution and specific surface area were characterized with laser diffraction method, using Analyzette 22 NanoTec (Fritsch GmbH, Idar-Oberstein, Germany). Powder morphology and ceramics fractured surfaces microstructure were studied by scanning electron microscopy (SEM), using JSM-6390LA (JEOL Ltd., Tokyo, Japan). The powders primary particle size, as well as ceramic samples grain size, were assessed by the line-intercept method from the SEM images. Phase analysis of the initial powder and crashed ceramic samples was conducted with Rigaku D/Max-2500 (Rigaku Corp., Tokyo, Japan) X-ray diffractometer. The patterns were recorded using CuKα radiation in a range of 10°< 2θ< 70° with a step of 0.02°. Phases were identified with the use of Crystallography Open Database (COD) [22]. GSAS software was applied for fitting of diffraction pattern profiles by Le Bail method and for the refinement of unit cell parameters by Rietveld method [23,24]. 

The ceramic samples in a shape of cylinders or rectangular prisms manufactured by traditional and additive methods, respectively, were polished to achieve plane and parallel bases and height of 1.5 ± 0.05 mm. During the polishing, the height of samples was controlled with the use of a frame. Then, the bases of the samples were metallized with silver paste and heated up to 800 °C for 15 min. Measurements of resistance, capacitance, and tangent of dielectric losses were carried out with the Immitance Meter RLC-781 05G (Good Will Instrument Co., Ltd., New Taipei City, Taiwan), at 1 kHz of AC frequency. The dielectric permittivity was calculated from the capacitance and geometrical parameters of samples. The piezoelectric module d_33_ was measured by a quasi-static method using the D33 Test Meter (Sinoceramics, Inc., Shanghai, China).

Density of ceramic samples was measured by Archimedes method. To study microhardness, BT ceramic samples were framed with epoxy pellets and polished with subsequent chemical etching by HNO_3_ water solution. Samples microhardness was measured with Nanovea PB1000 (Nanovea Inc., Irvine, CA, USA) analytical setup. Berkovich micro-indentor was used at the maximum load of 10 N. The loading rate was 1 N/min.

## 3. Results

### 3.1. Photopolymerization

**Figure 4:**
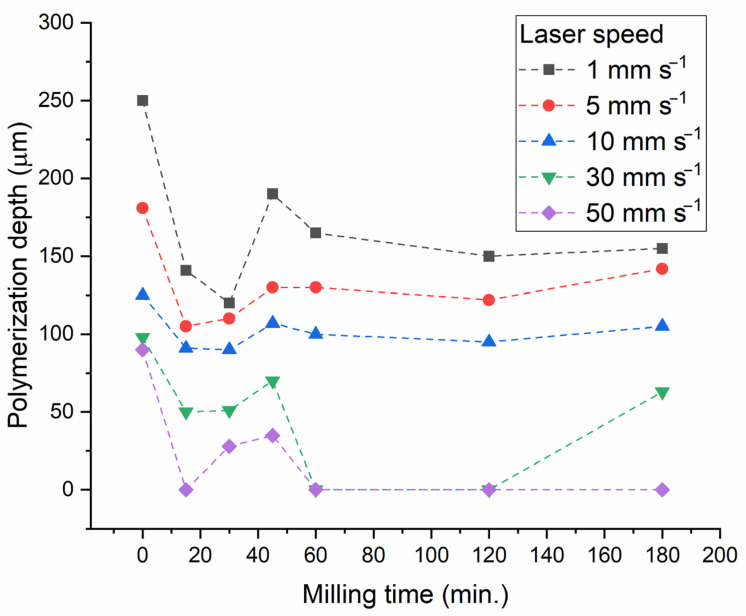
The photopolymerization depth of paste samples filled with powder with different milling times, depending on the laser scanning speed.

Each type of paste was used for experiments to determine the maximum photopolymerization depth and for 3D printing of piezoceramic samples (Figure 4). The photopolymerization testing was conducted at different speeds of laser motion with the robot. As a result, a greater or smaller amount of energy was deposited into the ceramic paste. The polymerization depth decreases with a decrease of the powder size due to more significant light scattering. After 60 min of milling at the scanning speed of 30 and 50 mm/s, photopolymerization stops. Only powders milled at 15–45 min (BT-2, BT-3 and BT-4) demonstrated photopolymerization at even high laser speed (up to 50 mm/s). The highest photopolymerization depth (>100 μm) was observed for the powder milled for 45 min. (BT-4), at laser speeds of 1–10 mm/s. An additional test for BT-4 paste was carried out with laser speed of 3 mm/s. This test indicated the polymerization depth of 146 μm, which was suitable for providing the interlock of 100 μm-thick paste layers in 3D-printing procedure. The layer thickness had to be selected thick enough (100 μm) because the laser speed was limited with the mechanically moving robot and the low power of the laser. The 3D-printing of thick items could be finished within a reasonable time when thick layers were used. The parameters selected for 3D printing of thick samples were as follows: hatching spacing 0.2 mm, laser speed 3 mm/s, and layer thickness 100 μm.

### 3.2. Materials Characterization

**Figure 5:**
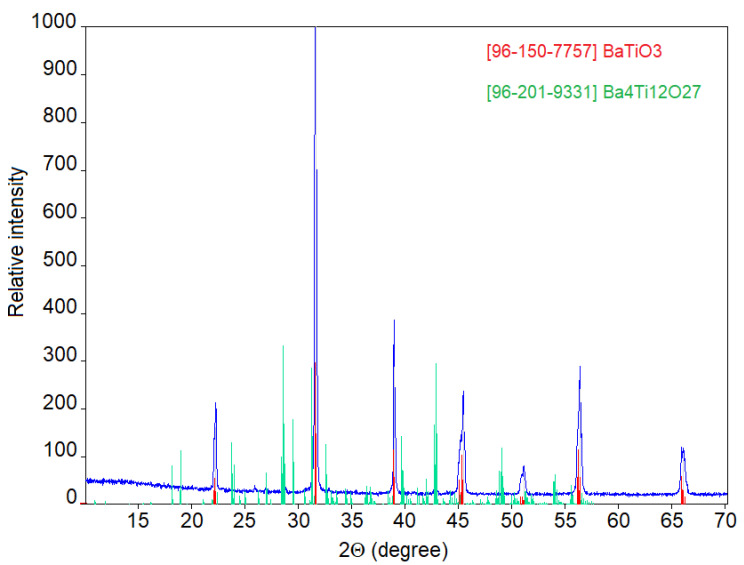
XRD pattern of the commercial BT powder.

The XRD pattern of commercial BT powder is shown in Figure 5. The observed peaks were indexed for tetragonal crystal structure in a good agreement with the reported data (COD #96-150-7757). The calculator cell parameters were a = b = 3.9925 Å, c = 4.0183 Å, BT tetragonality c/a = 1.0065. Besides the peaks of this main phase, minor peaks of polytitanate Ba_4_Ti_12_O_27_ were distinguished, as well (COD #96-201-9331).

**Figure 6:**
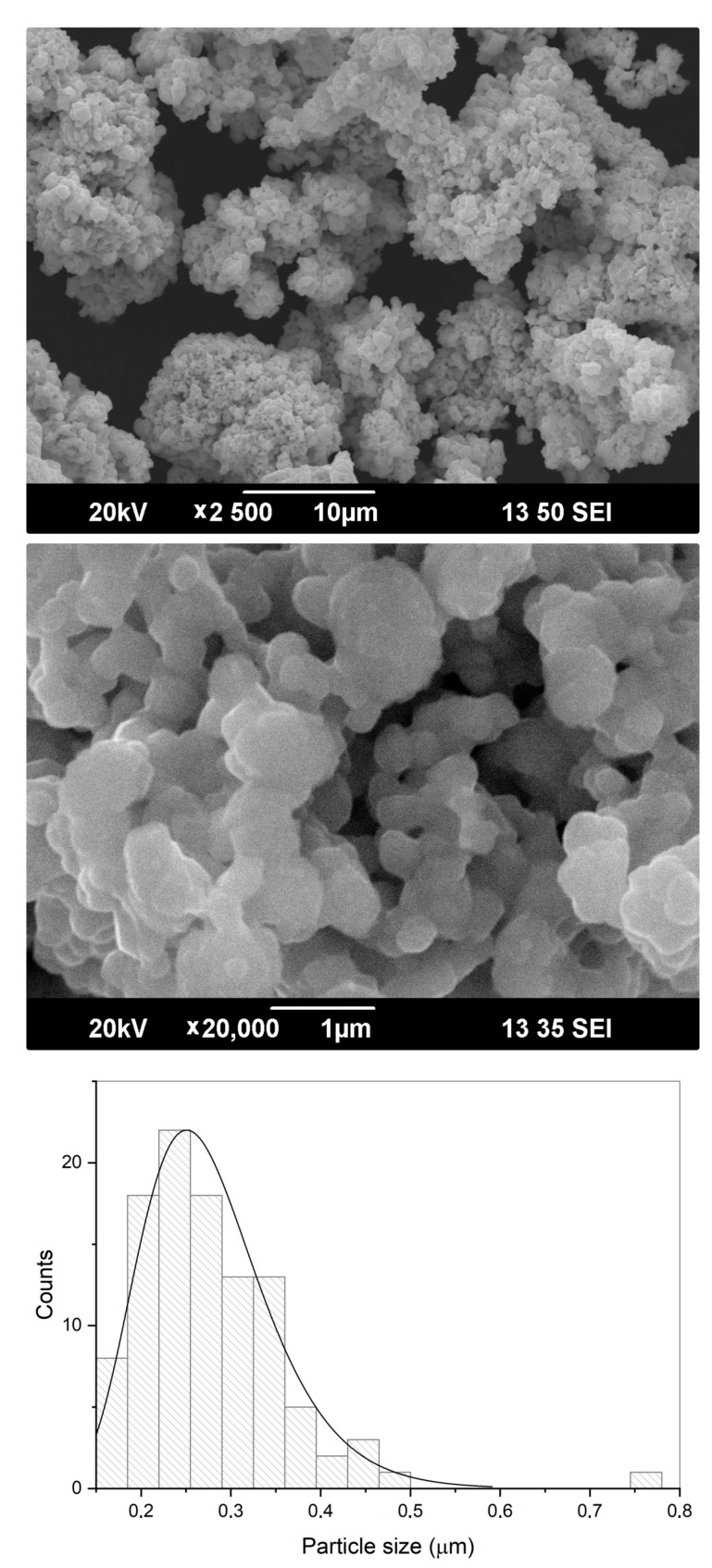
Morphological characteristics of the commercial BT powder: SEM micrographs and primary particle size distribution from SEM data.

**Figure 7:**
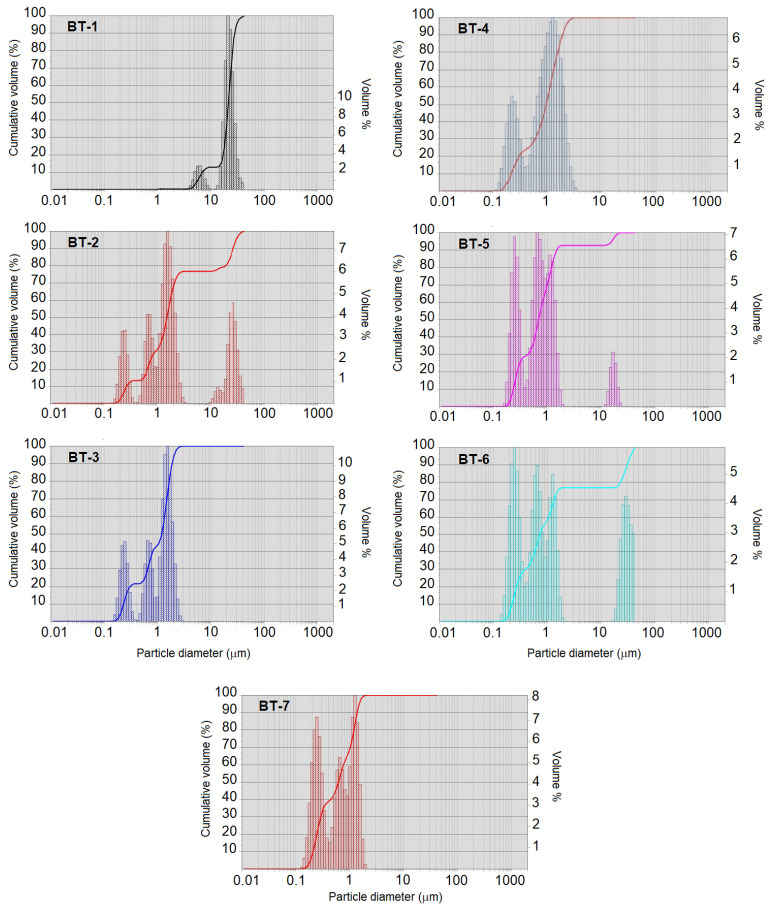
Laser diffraction data for the commercial BT powder before and after milling procedure (sample names referred to Table 1).

SEM micrographs shown in Figure 6, in conjunction with the results of laser diffraction study (Table 2, Figure 7), revealed that BT powder consisted of agglomerates with bimodal size distribution characterized by mean values of 1.2 and 21.5 μm for minor and major fractions, respectively. The primary particles which formed these agglomerates had rounded shape and were measured in a range of 160–750 nm with a mean size of 279 nm.

The milling procedure was rather effective in large agglomerates’ destruction (Table 2, Figure 7). Both minor and major fractions found in the initial powder (BT-1) gradually disappeared during the first 30 min of milling (samples BT-2 and BT-3 were milled for 15 and 30 min, respectively). They were replaced by two or three fractions in a range of 0.20–1.25 μm. The finer fraction corresponded well to the size range of primary particles in the initial powder. The milling prolonged to 60 and 120 min (corresponding to the samples BT-5 and BT-6) led to particle re-agglomeration and appearance of a fraction over 10 μm. However, further milling (BT-7 was treated for 180 min) resulted in coarse agglomerates destruction.

**Figure 8:**
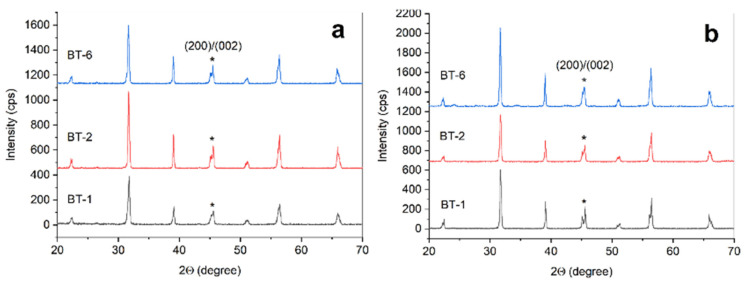
XRD patterns of ceramic samples prepared by additive (**a**) and conventional (**b**) manufacturing. Patterns captions indicate the type of source powder. *—peaks at about 45°2θ.

**Figure 9:**
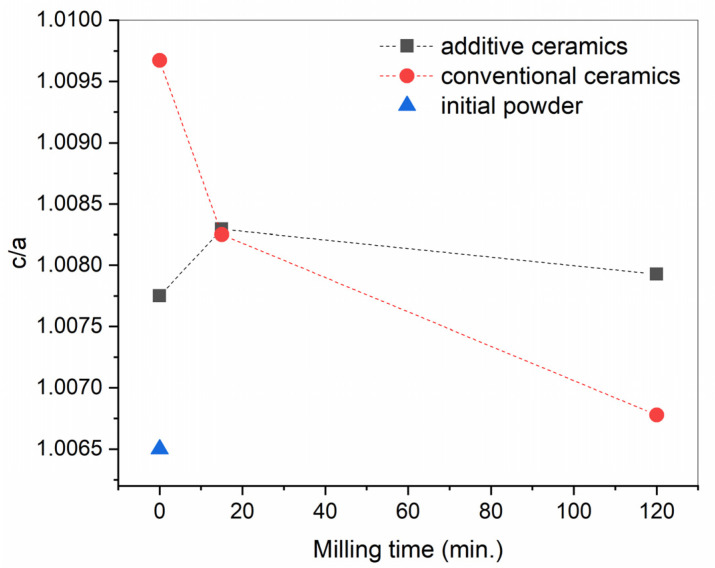
Ratio of cell parameters c/a calculated for tetragonal BT ceramics prepared by conventional and additive manufacturing.

Peak positions observed in XRD patterns of the ceramic samples prepared from the commercial BT powder by traditional and additive manufacturing routes corresponded to tetragonal modification of BaTiO_3_, COD #96-150-7757 (Figure 8). At visual estimation, splitting of the peaks at about 45°2θ inherent in tetragonal BT significantly differed between conventionally-produced samples. In the traditionally prepared series, the prolongation of powder milling was accompanied by the descending c/a ratio in final ceramics (Figure 9). In the printed series of ceramics, the tetragonality performed no obvious correlation with the duration of milling and varied less between the examined samples. However, in both series of ceramics, the sintering procedure led to an increase in tetragonality compared to the initial BT powder.

**Figure 10:**
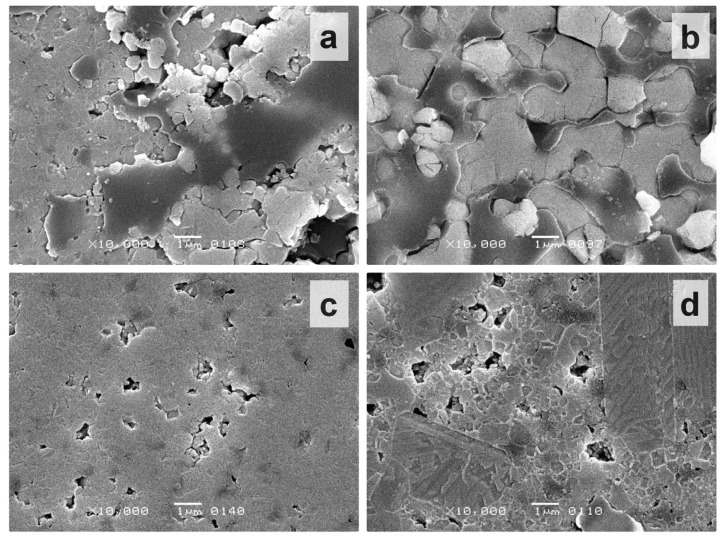
SEM images of polished surface after chemical etching: (**a**) additive manufacturing, without milling of initial BT powder; (**b**) conventional manufacturing, without milling of initial BT powder; (**c**) additive manufacturing, 30 min of initial powder milling; (**d**) conventional manufacturing, 30 min of initial powder milling.

Variations in tetragonality of ceramics originated from its microstructure. Typical microstructures of the BT ceramics obtained with additive and traditional procedures are shown in Figure 10. The highest c/a ratio corresponded to the material with micron-sized grains (Figure 10b, mean grain size of 1.86 ± 0.57 μm). Milling of the initial BT powder, as well as the manufacturing method, strongly influenced the microstructure of the samples. A common feature of the ceramics prepared from the non-milled powder was the presence of smoothed areas of liquid phase formed during the sintering (Figure 10a,b). These areas coexisted with the regions of compacted grains. Liquid phase formation was caused by TiO_2_ excess in relation to BaO in the initial BT powder (small amounts of Ba_4_Ti_12_O_27_ detected by XRD). The sintering temperature of 1300 °C was close to the eutectic value of BaTiO_3_–Ba_6_Ti_17_O_40_ (about 1320 °C) [25,26]. After the milling of powder, the traces of liquid phase disappeared (Figure 10c,d). Additive manufacturing resulted in a material with relatively homogenous fine-grained microstructure (Figure 10c). The mean size of grains slightly varied between the samples in a range of 440–510 nm. In the traditionally prepared ceramics, a distinct growth of abnormal grains (AG) was observed (Figure 10d) and was accompanied by a decrease in tetragonality. Table 3 demonstrates the sizes of AG in the samples sintered after 30, 45, and 180 min of powder milling. The mean size of AG increased with increasing powder milling time. It is peculiar to the abnormal grain growth that large grains are surrounded by a low-density fine-grained matrix [27,28]. Volume polarization in these low-density areas is hindered. For this reason, the decrease of tetragonality was observed in the conventionally-manufactured samples with prolongation of milling (Figure 9). Slightly elongated AG are likely to grow below the eutectic mentioned above, while, above the eutectic, they appear equiaxed [25]. TiO_2_ excess in the raw BT powder is often assumed as a condition for AG growth [26]. However, the microstructure of the 3D-printed ceramics indicated that TiO_2_ excess in the initial BT powder was necessary but not sufficient for AG growth (Figure 10c). Local strains occurring on green body pressing could be assumed as a supplementary factor for the formation of AG [29,30,31]. In a case of 3D printing, the powder particles underwent a slow packing process during the debinding, without any external mechanical pressure. Probably, for this reason, AG growth was not notable in the 3D-printed ceramics.

### 3.3. Ceramics Properties

Piezoelectric properties, relative density, and microhardness for additive manufactured and traditional samples obtained with pressing are presented in Table 4 and Table 5, respectively.

#### 3.3.1. Relative Density

**Figure 11:**
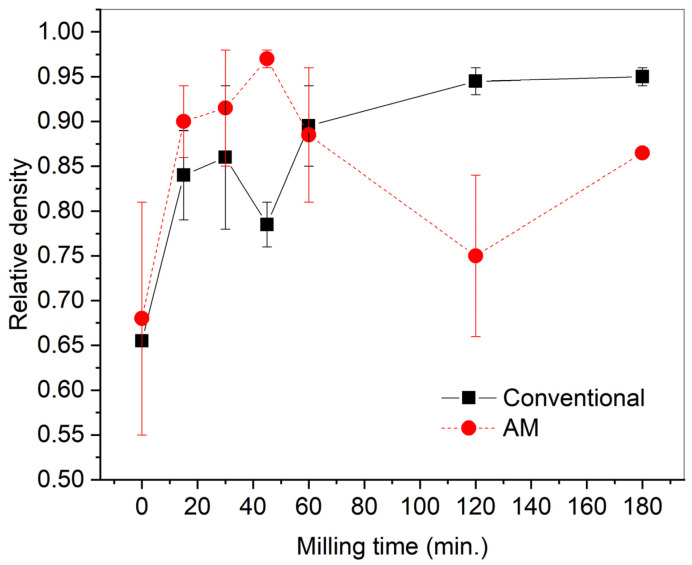
Relative density versus powder milling time for 3D-printed and conventional samples.

The relative density of sintered BT ceramic samples varies significantly due to the large difference in particle sizes and morphology of the initial powders, for both additively manufactured samples and samples obtained with pressing (Figure 11). For samples obtained with pressing, there is a tendency to increase the relative density up to 0.95 with increasing milling time. The growth of abnormal grains is known to limit densification because of mass transfer preferentially occurring from fine to coarse grains [27]. For 3D-printed samples, the highest relative density (over 0.95) was achieved when using powders with the 30- and 45-min milling time (BT-3 and BT-4). Further milling performed a reverse effect on the density because of particle re-agglomeration (Figure 7) and decrease in photopolymerization depth (Figure 4). 

#### 3.3.2. Piezoelectric Properties

**Figure 12:**
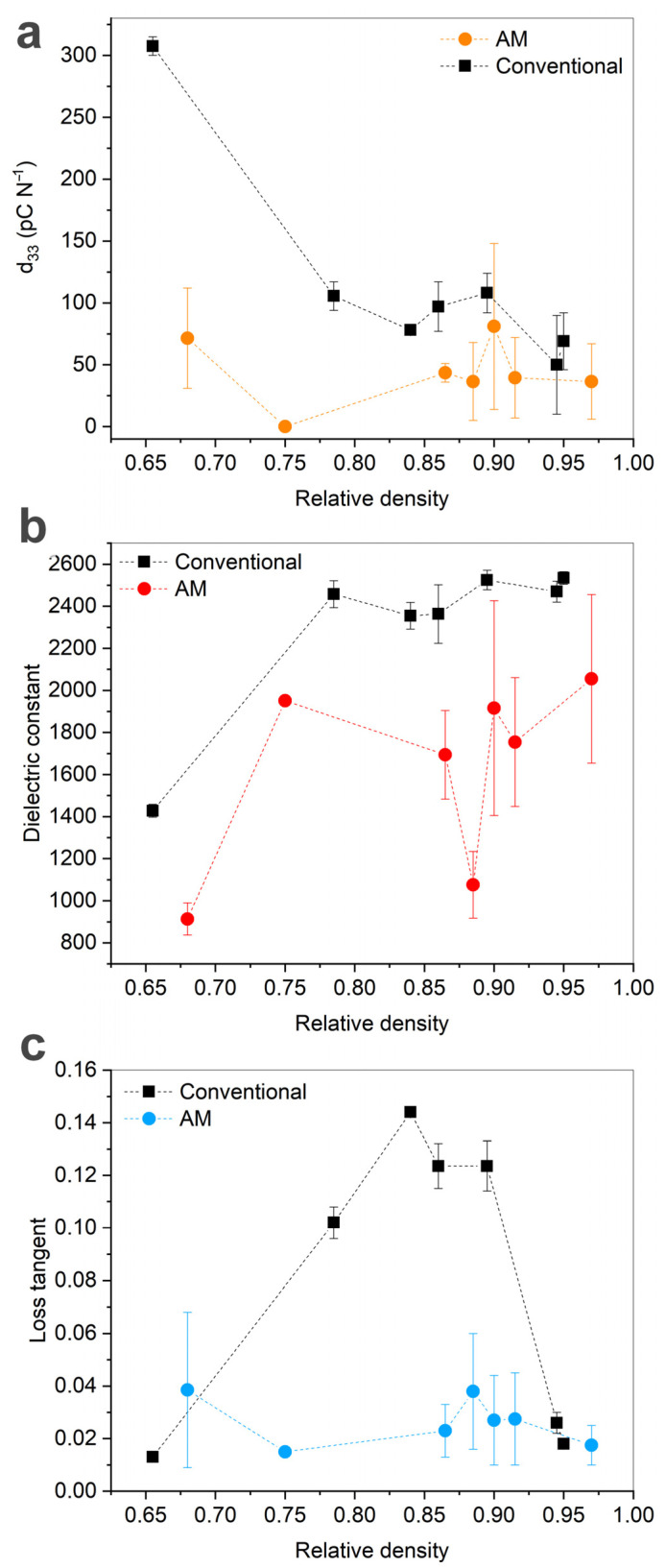
Relative density and piezoelectric properties for 3D-printed and conventional samples: (**a**) piezo module d_33_; (**b**) dielectric constant ε; (**c**) loss tangent tgδ.

The values of the piezo module d_33_ of 3D-printed and traditionally prepared ceramics demonstrated complex behavior with the changes in the relative density (Figure 12). The additive and conventional samples of the highest density did not demonstrate the maximum values of the piezo module d_33_. For the 3D-printed samples, the maximum d_33_ = 148 pC/N was achieved in the BT-2-AM2 sample, with a relative density of 0.86. Among the samples obtained by pressing, the maximum d_33_ = 305 pC/N was registered in the BT-1-P1 sample, with a small relative density of 0.66. While the printed ceramics showed no obvious dependence of d_33_ on the relative density, the traditional samples showed an increase in d_33_ with reducing density. This result might be caused by intensification of flex-tensional deformations of the solid surrounded by the porous space under mechanical or electric influence [32]. 

**Figure 13:**
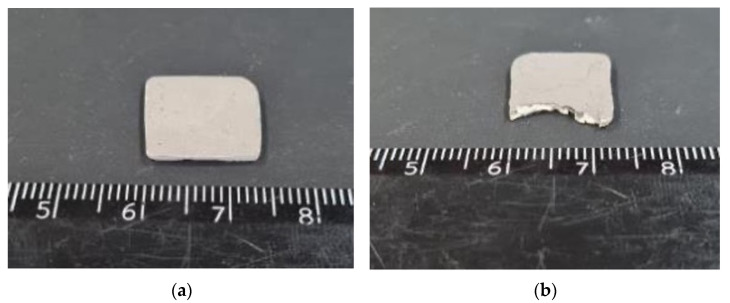
Three-dimensional-printed and sintered BT ceramic samples after metallization: (**a**) BT-2-AM2 (d_33_ = 148 pC/N); (**b**) BT4-AM2. The BT-4-AM2 sample is not suitable for polarization due to the penetration of silver paste with through cracks.

The permittivity (measured at 1 kHz and room temperature) demonstrated values of 2355–2535 for traditional ceramics and 1695–2055 for the 3D-printed ones, with an exception of the most porous samples with low ε (Figure 12b). Besides, the graph for the printed samples showed large deviations and a significant decrease in the dielectric constant for the powder with the 60 min (BT-5) milling time due to the penetration of silver electrode paste into macrostructural inhomogeneities (i.e., cracks and large pores) during metallization (Figure 13). The loss tangent of the printed ceramics showed no dependence on the relative density and did not exceed 0.04. Conventionally-prepared materials with the relative density of 0.78–0.88 had high losses of 0.10–0.15, while other samples also possessed tgδ < 0.026. These high losses could be attributed to the influence of moisture accumulated in open pores among the loose fine-grained matrix. With increasing porosity and internal surface, the domain walls fixation led to a decrease in loss tangent [32].

#### 3.3.3. Microhardness

**Figure 14:**
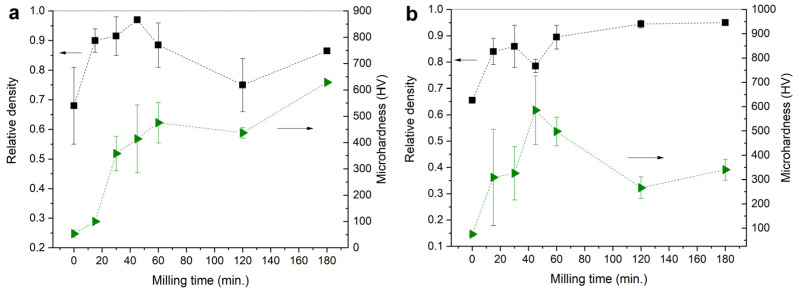
Relative density and microhardness for: (**a**) 3D-printed samples; (**b**) conventional samples.

Microhardness of 3D-printed ceramics showed an obvious correlation with their relative density due to rather high microstructural homogeneity (Figure 14a). A similar correlation was observed among traditional ceramics prepared from powders with short milling time (0–30 min) (Figure 14b). In the cases of longer milling time (45–180 min), the microhardness decreased with the powder milling time increase, even though the relative density showed a slow increase. This effect should be attributed to the intensification of AG growth, which led to formation of loose fine-grained areas with low hardness.

## 4. Discussion

The results described above showed that SLA technique with a wavelength of 465 nm allows manufacturing of BT ceramics with adequate phase, microstructural, piezoelectric, and mechanical properties from a commercially available powder with a micron particle size, which is commonly used in large-scale industrial manufacturing of different BT ceramic products. A comparison with the materials prepared in parallel by the traditional ceramic route revealed a great potential of additive manufacturing approach. In both preparation methods, rather high density (over 95%), as well as tetragonality, of BT ceramics was achieved, promising for satisfactory functional characteristics. The 3D printing technique appeared advantageous over the traditional one in suppressing of the undesired AG growth in TiO_2_-excessive matrix due to the difference in green body compaction. This feature resulted in predictable relation of microhardness and density, as well as in stable and comparatively low dielectric losses. Along with piezo module d_33_ and dielectric constant ε, the dissipation factor tgδ is sensitive to microstructure of piezoceramics and determines their suitability for any application [33,34]. Low dielectric losses are the desired characteristics for most of piezoceramics applications. In the current work, the complex of piezoelectric properties performed by the 3D-printed materials with the relative density over 75% (d_33_ of 36.5–148.0 pC/N, ε of 1695–2055, and tgδ of 0.018–0.028) appeared comparable to that of the conventionally-manufactured samples (d_33_ of 50.0–105.0 pC/N, ε of 2355–2525, and tgδ of 0.018–0.144), if not taking into account conventional low-density samples, fabricated from non-milled powder. Comparatively high dielectric permittivity of the conventional samples could be attributed to the conductivity which generated the increased losses, as well.

**Figure 15:**
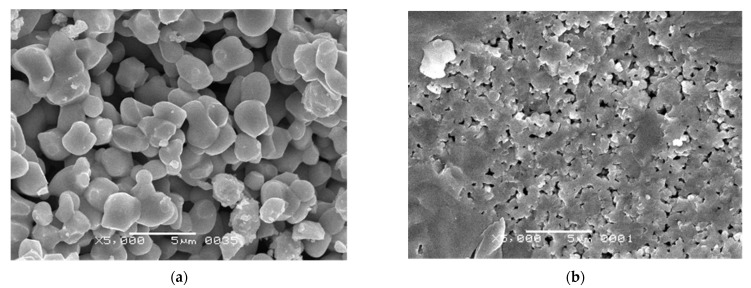
The fractured surface microstructure of the sintered conventional manufactured samples: (**a**) BT-1-P1 (d_33_ = 315 pC/N); (**b**) BT-6-P1 (d_33_ = 90 pC/N).

The microstructure analysis showed that the conventionally-manufactured samples from the initial non-milled powder differ markedly in grain size and relative density from other samples. The distinctive features of these microstructures are small grains (on average 1–3 microns), tightly sintered into a porous frame. The reason for this is probably the morphology of the initial powder, which is dense sintered agglomerates of particles in sub-micron size (Figure 6 and Figure 7). After compaction by pressing, such agglomerates do not allow dense packing of grains, and grain growth during the sintering is limited (Figure 15a). Grains of this size in BT ceramics [35] provide maximum values of the piezo module d_33_, exceeding 300 pC/N, and also may be caused by intensification of flex-tensional deformations [32]. This can explain the significant difference in the piezo module of the samples studied in this work. In addition, unlike the microstructures of the BT-6-P1 sample (Figure 15b), which has a piezo module of d_33_ = 90 pC/N, there is no abnormal grain growth that reduces piezoelectric properties, despite the high density of ceramics. On the other hand, milling of the initial powder, which has an excess of TiO_2_ in relation to BaO, leads to a denser packing of particles during compaction and intensive mass transfer during sintering, which provokes AG growth.

**Figure 16:**
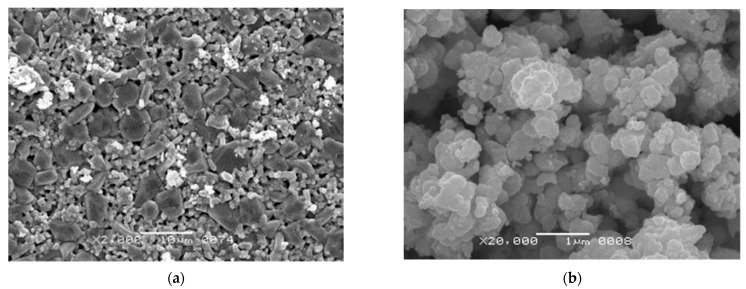
The SEM images of: (**a**) fractured surface microstructure of the sintered 3D-printed sample BT-1-AM2 (d_33_ = 37 pC/N); (**b**) debinded powder after high-speed mixing with a solution of oligomers and photo initiators.

Comparison of microstructures of samples fractured surfaces from non-milled powder obtained with pressing BT-1-P1 (piezo module d_33_ = 315 pC/N) (Figure 15a) and 3D printing (sample BT-1-AM2), piezo module d_33_ = 37 pC/N (Figure 16a), shows a pronounced difference in density and grain sizes. It can be explained by the technological feature of the paste preparation for SLA 3D printing operations used in this study. The powder is mixed with an organic binder in a planetary mill. Due to the mixing, the initial agglomerates of the non-milled powder were partially broken (Figure 16b), fine particles were tightly packed, and AG growth occurred, mainly oriented along with the photopolymerization layers. Probably, the approach of using a large particle size powder consisting of dense agglomerates with a size of about 1 micron could be successfully applied in the piezoceramics SLA 3D printing if the methods of paste preparation without high-energy mixing are applied. However, this assumption requires detailed research.

In addition to the difference in microstructure and properties between additive and conventionally-manufactured samples, there is a pronounced difference in the dependence of the relative density on the milling time and, accordingly, on the size and specific surface area of the powder particles (Figure 11). For conventionally-pressed samples, the dependence is usual, i.e., with an increase in the milling time and a decrease in the size of powder particles, the relative density of sintered ceramic samples increases. For 3D-printed samples, a local maximum of relative density is observed at 45 min of powder milling, after which the relative density decreases. This regularity correlates with the local maximum photopolymerization depth achieved with the powder milled for 45 min. Probably, for the photopolymer composition used in this study, the characteristics of BT-4 powder (d_50_ = 0.93 μm, specific surface area 110,538 cm^2^/cm^3^) (Table 2) are the most suitable. A further decrease in particle size and an increase in the specific surface area leads to a deterioration of photopolymerization, probably due to intense light scattering. The use of BT powder in the SLA 3D printing at a wavelength of 465 nm requires a certain composition and concentration of the photoinitiator for particles of different sizes and different specific surface areas.

Comparison of this work results with the available data on the BT ceramics SLA 3D printing process features and the achieved piezoelectric properties (Table 6) shows that using a 465 nm wavelength light source can significantly increase the photopolymerization depth of more than 50 vol.% BT paste without any adverse effects of the layers’ delamination during printing. In addition, the most considerable value of the piezo modulus, d_33_ = 148 pC /N, was achieved using a micron BT powder with a mean particle size of 1.5 μm, which is more practically applicable in comparison with submicron and nanoscale powders used in the studies of other authors. Finally, measurements of the microhardness of additive and traditional samples made it possible to conduct the first preliminary assessment of the level of mechanical properties of BT piezoceramics, not previously presented in the literature.

## 5. Conclusions

In the current work, barium titanate ceramics were manufactured by both conventional and additive manufacturing methods from the same powders of different dispersity using identical sintering conditions. In both methods, high relative density of ceramics over 0.95 was achieved. SLA technique appeared advantageous over the traditional one in suppressing a typical and undesired phenomenon of abnormal grain (AG) growth occurring in the TiO_2_-excessive matrix due to the difference in green body compaction. This feature resulted in predictable relation of microhardness (over 500 HV) and relative density, as well as stable and comparatively low dielectric losses tgδ. The complex of piezoelectric properties performed by the 3D-printed barium titanate ceramics with the relative density over 75% (d_33_ of 36.5–148.0 pC/N, ε of 1695–2055, and tgδ of 0.018–0.028) appeared comparable to that of the conventionally-manufactured samples (d_33_ of 50.0–105.0 pC/N, ε of 2355–2525, and tgδ of 0.018–0.144). The present study demonstrated the relatively low-cost way for SLA 3D printing at 465 nm wavelength of barium titanate piezoelectric ceramics using micron-sized powders and high printed layer thickness (over 100 μm). The production of barium titanate ceramics via this technique could be further optimized.

## Figures and Tables

**Table 1:** Powder volume fraction of ceramic pastes.

Powder Sample	Milling Time, Min	Powder Volume Fraction, %
BT-1	0	37.14
BT-2	15	52.33
BT-3	30	51.24
BT-4	45	50.10
BT-5	60	49.83
BT-6	120	42.38
BT-7	180	51.82

**Table 2:** BT milled powders particle size distribution and specific surface area.

Powder Sample	Milling Time, Min	Modal Diameter, μm	d_90_, μm	d_50_, μm	d_10_, μm	Specific Surface Area, cm²/cm³
BT-1	0	20.82	26.94	19.96	9.42	3855
BT-2	15	1.53	26.21	1.5	0.26	68,591
BT-3	30	1.49	1.89	1.2	0.23	97,836
BT-4	45	1.27	1.85	0.93	0.21	110,538
BT-5	60	0.67	1.48	0.68	0.23	118,969
BT-6	120	0.24	1.67	0.72	0.22	112,420
BT-7	180	1.23	1.37	0.61	0.21	141,825

**Table 3:** Characteristics of abnormal grains in conventionally-prepared BT ceramics.

Duration of the Initial BT Powder Milling, Min	Length of Abnormal Grain, μm			Mean Length to Width Ratio
	Minimum	Maximum	Mean	
30	2.7	50.8	9.3 ± 5.1	2.0 ± 1.0
45	4.4	21.5	11.0 ± 3.7	2.3 ± 1.3
80	5.0	25.8	13.6 ± 4.7	1.7 ± 0.5

**Table 4:** Properties of additively manufactured samples.

Powder Sample	Piezoceramic Sample Code	ε (1 kHz)	tgδ (1 kHz)	d33, pC/N	Microhardness, HV	Relative Density
BT-1	BT-1-AM1	837	0.061	31	39.77	0.62
	BT-1-AM2	857	0.068	37	44.63	0.55
	BT-1-AM3	990	0.009	112	67.79	0.81
BT-2	BT-2-AM1	2006	0.037	52	104.05	0.88
	BT-2-AM2	1405	0.044	148	96.11	0.86
	BT-2-AM3	2426	0.01	14	-	0.94
BT-3	BT-3-AM1	1499	0.045	72	292.24	0.85
	BT-3-AM2	2061	0.033	7	357.01	0.98
	BT-3-AM3	1448	0.01	8	424.04	0.94
BT-4	BT-4-AM1	1654	0.025	67	285.42	0.97
	BT-4-AM2	0	-	0	317.78	0.98
	BT-4-AM3	2456	0.01	6	543.26	0.96
BT-5	BT-5-AM1	1234	0.06	68	420.26	0.81
	BT-5-AM2	0	-	0	398.30	0.96
	BT-5-AM3	918	0.016	5	552.66	0.95
BT-6	BT-6-AM1	0	-	0	416.94	0.84
	BT-6-AM2	0	-	0	442.75	0.66
	BT-6-AM3	1951	0.015	0	456.94	0.80
BT-7	BT-7-AM1	1531	0.033	51	-	0.86
	BT-7-AM2	1905	0.013	0	628.84	0.87
	BT-7-AM3	1484	0.026	36	-	0.86

**Table 5:** Properties of conventionally-manufactured samples (pressing).

Powder Sample	Piezoceramic Sample Code	ε (1 kHz)	tgδ (1 kHz)	d33, pC/N	Microhardness, HV	Relative Density
BT-1	BT-1-P1	1459	0.013	315	65.59	0.66
	BT-1-P2	1397	0.013	300	84.99	0.65
	BT-1-P3	1324	0.012	280	78.73	0.62
BT-2	BT-2-P1	2418	0.143	81	194.88	0.79
	BT-2-P2	2394	0.145	80	507.01	0.89
	BT-2-P3	2292	0.144	75	110.68	0.83
BT-3	BT-3-P1	2387	0.119	117	215.99	0.94
	BT-3-P2	2502	0.132	77	430.89	0.93
	BT-3-P3	2224	0.115	78	435.76	0.78
BT-4	BT-4-P1	2394	0.096	94	444.21	0.80
	BT-4-P2	2423	0.098	105	726.51	0.81
	BT-4-P3	2521	0.108	117	493.99	0.76
BT-5	BT-5-P1	2478	0.114	124	438.74	0.94
	BT-5-P2	2516	0.117	92	556.91	0.85
	BT-5-P3	2571	0.133	117	538.61	0.94
BT-6	BT-6-P1	2420	0.022	90	222.06	0.96
	BT-6-P2	2519	0.025	11	310.85	0.93
	BT-6-P3	2484	0.030	10	297.34	0.95
BT-7	BT-7-P1	2534	0.019	92	297.95	0.94
	BT-7-P2	2504	0.018	60	336.99	0.96
	BT-7-P3	2565	0.017	46	383.54	0.95

**Table 6:** Comparison of the experimental results of BT ceramics 3D printing by SLA method.

#	Mean Powder Particle Size, μm	Light Wavelength, nm	Photopolymerization Depth, μm	d_33_, pC/N	tan δ	ε	Micro Hardness, HV	Reference
1	0.5	-	-	166	0.036	2177	-	[4]
2	0.428	405	90 (40 vol.% BT)	163	0.016	2762	-	[19]
3	1.5	465	>120 (52 vol.% BT)	148	0.044	1405	96.11	This work

## Data Availability

The data presented in this study are available on request from the corresponding author after obtaining the permission of an authorized person.
